# Spatiotemporal variation in risk of *Shigella* infection in childhood: a global risk mapping and prediction model using individual participant data

**DOI:** 10.1016/S2214-109X(22)00549-6

**Published:** 2023-02-14

**Authors:** Hamada S Badr, Josh M Colston, Nhat-Lan H Nguyen, Yen Ting Chen, Eleanor Burnett, Syed Asad Ali, Ajit Rayamajhi, Syed M Satter, Nguyen Van Trang, Daniel Eibach, Ralf Krumkamp, Jürgen May, Ayola Akim Adegnika, Gédéon Prince Manouana, Peter Gottfried Kremsner, Roma Chilengi, Luiza Hatyoka, Amanda K Debes, Jerome Ateudjieu, Abu S G Faruque, M Jahangir Hossain, Suman Kanungo, Karen L Kotloff, Inácio Mandomando, M Imran Nisar, Richard Omore, Samba O Sow, Anita K M Zaidi, Nathalie Lambrecht, Bright Adu, Nicola Page, James A Platts-Mills, Cesar Mavacala Freitas, Tuula Pelkonen, Per Ashorn, Kenneth Maleta, Tahmeed Ahmed, Pascal Bessong, Zulfiqar A Bhutta, Carl Mason, Estomih Mduma, Maribel P Olortegui, Pablo Peñataro Yori, Aldo A M Lima, Gagandeep Kang, Jean Humphrey, Robert Ntozini, Andrew J Prendergast, Kazuhisa Okada, Warawan Wongboot, Nina Langeland, Sabrina J Moyo, James Gaensbauer, Mario Melgar, Matthew Freeman, Anna N Chard, Vonethalom Thongpaseuth, Eric Houpt, Benjamin F Zaitchik, Margaret N Kosek

**Affiliations:** a Department of Earth and Planetary Sciences, Johns Hopkins Krieger School of Arts and Sciences, Baltimore, MA, USA; b Division of Infectious Diseases and International Health, University of Virginia School of Medicine, Charlottesville, VA, USA; c College of Arts and Sciences, University of Virginia, VI, USA; d Department of Emergency Medicine, Chi-Mei Medical Center, Tainan, Taiwan; e Division of Viral Diseases, US Centers for Disease Control and Prevention, Atlanta, GA, USA; f Department of Pediatrics and Child Health, Aga Khan University, Karachi, Pakistan; g Center of Excellence in Women and Child Health, Aga Khan University, Karachi, Pakistan; h Department of Pediatrics, National Academy of Medical Sciences, Kanti Children's Hospital, Kathmandu, Nepal; i Programme for Emerging Infections, Infectious Diseases Division, International Centre for Diarrhoeal Disease Research, Bangladesh (icddr,b), Dhaka, Bangladesh; j Centre for Nutrition & Food Security, International Centre for Diarrhoeal Disease Research, Bangladesh (icddr,b), Dhaka, Bangladesh; k Nutrition and Clinical Services Division, International Centre for Diarrhoeal Disease Research, Bangladesh (icddr,b), Dhaka, Bangladesh; l National Institute of Hygiene and Epidemiology, Ha Noi, Vietnam; m Department of Infectious Disease Epidemiology, Bernhard Nocht Institute for Tropical Medicine (BNITM), Hamburg, Germany; n Institute of Tropical Medicine, Universitätsklinikum Tübingen, Tübingen, Germany; o Centre for Infectious Disease Research in Zambia, Lusaka, Zambia; p Enteric diseases and Vaccines Unit, Centre for Infectious Disease Research in Zambia, Lusaka, Zambia; q Department of International Health, Johns Hopkins Bloomberg School of Public Health, Baltimore, MD, USA; r Faculty of Medicine and Pharmaceutical Sciences, University of Dschang, Dschang, Cameroon; s Department of Health Research, M A SANTE (Meileur Acces aux Soins en Santé), Yaoundé, Cameroon; t Division of Health Operations Research, Cameroon Ministry of Public Health, Yaoundé, Cameroon; u Medical Research Council Unit, The Gambia at the London School of Hygiene & Tropical Medicine, Banjul, The Gambia; v National Institute of Cholera and Enteric Diseases, Kolkota, India; w Department of Pediatrics, University of Maryland School of Medicine, Baltimore, MD, USA; x Centro de Investigação em Saúde de Manhiça, Manhica, Mozambique; y Kenya Medical Research Institute, Center for Global Health Research, Kisumu, Nyanza, Kenya; z Centre pour le Développement des Vaccins, Mali, Bamako, Mali; aa Institute of Public Health, Charité - Universitätsmedizin Berlin, Corporate Member of Freie Universität Berlin and Humboldt-Universität zu Berlin, Berlin, Germany; ab Research Department 2, Potsdam Institute for Climate Impact Research (PIK), Member of the Leibniz Association, Potsdam, Germany; ac Department of Immunology, Noguchi Memorial Institute for Medical Research, University of Ghana, Legon, Ghana; ad Centre for Enteric Diseases, National Institute for Communicable Diseases, Pretoria, South Africa; ae Hospital Pediátrico David Bernardino, Luanda, Angola; af New Children's Hospital, Pediatric Research Center and Helsinki University Hospital, Helsinki, Finland; ag Centre for Child, Adolescent, and Maternal Health Research, Faculty of Medicine and Health Technology, Tampere University and Tampere University Hospital, Tampere, Finland; ah College of Medicine, University of Malawi, Blantyre, Malawi; ai HIV/AIDS & Global Health Research Programme, University of Venda, Thohoyandou, Limpopo, South Africa; aj Department of Enteric Diseases, Armed Forces Research Institute of Medical Sciences (AFRIMS), Bangkok, Thailand; ak Haydom Global Health Institute, Haydom, Tanzania; al Asociacion Benefica PRISMA, Iquitos, Peru; am Department of Physiology and Pharmacology, Faculty of Medicine, Federal University of Ceará, Fortaleza, Brazil; an Department of Gastrointestinal Sciences, Christian Medical College, Vellore, India; ao Zvitambo Institute for Maternal and Child Health Research, Harare, Zimbabwe; ap Blizard Institute, Queen Mary University of London, London, UK; aq Research Institute for Microbial Diseases, Osaka University, Osaka, Japan; ar Department of Medical Sciences, National Institute of Health, Nonthaburi, Thailand; as Department of Clinical Science, University of Bergen, Bergen, Norway; at Center for Global Health, Department of Epidemiology, Colorado School of Public Health, Aurora, CO, USA; au Pediatric Infectious Diseases, Hospital Roosevelt, Guatemala City, Guatemala; av Gangarosa Department of Environmental Health, Rollins School of Public Health, Emory University, Atlanta, 30322, GA, USA; aw Laboratory and Treatment Unit, Center for Malariology, Parasitology, and Entomology, Ministry of Health, Vientiane, Lao PDR

## Abstract

**Background:**

Diarrhoeal disease is a leading cause of childhood illness and death globally, and *Shigella* is a major aetiological contributor for which a vaccine might soon be available. The primary objective of this study was to model the spatiotemporal variation in paediatric *Shigella* infection and map its predicted prevalence across low-income and middle-income countries (LMICs).

**Methods:**

Individual participant data for *Shigella* positivity in stool samples were sourced from multiple LMIC-based studies of children aged 59 months or younger. Covariates included household-level and participant-level factors ascertained by study investigators and environmental and hydrometeorological variables extracted from various data products at georeferenced child locations. Multivariate models were fitted and prevalence predictions obtained by syndrome and age stratum.

**Findings:**

20 studies from 23 countries (including locations in Central America and South America, sub-Saharan Africa, and south and southeast Asia) contributed 66 563 sample results. Age, symptom status, and study design contributed most to model performance followed by temperature, wind speed, relative humidity, and soil moisture. Probability of *Shigella* infection exceeded 20% when both precipitation and soil moisture were above average and had a 43% peak in uncomplicated diarrhoea cases at 33°C temperatures, above which it decreased. Compared with unimproved sanitation, improved sanitation decreased the odds of *Shigella* infection by 19% (odds ratio [OR]=0·81 [95% CI 0·76–0·86]) and open defecation decreased them by 18% (OR=0·82 [0·76–0·88]).

**Interpretation:**

The distribution of *Shigella* is more sensitive to climatological factors, such as temperature, than previously recognised. Conditions in much of sub-Saharan Africa are particularly propitious for *Shigella* transmission, although hotspots also occur in South America and Central America, the Ganges–Brahmaputra Delta, and the island of New Guinea. These findings can inform prioritisation of populations for future vaccine trials and campaigns.

**Funding:**

NASA, National Institutes of Health–The National Institute of Allergy and Infectious Diseases, and Bill & Melinda Gates Foundation.

## Introduction

*Shigella*, a genus of gram-negative bacteria, infects over 267 million people annually in low-income and middle-income countries (LMICs) and is responsible for over 212 000 diarrhoeal disease deaths per year in all ages.^1^ It is most commonly recognised via its manifestation as bacillary dysentery, with 64 000 such deaths occurring in children younger than 5 years every year.^1^ *Shigella* transmission is particularly common in rural areas and in areas where environmental risk factors such as poor water quality, lack of access to care, and inadequate sanitation are prevalent.^1^, ^2^, ^3^ It is also associated with temperature^4^ and is sensitive to other meteorological conditions^5^ and events such as flooding.^6^ Vaccine development for *Shigella* has been hampered by biotechnical and financial limitations, but with multiple vaccine candidates now in late stages of development, it is becoming increasingly important to estimate the geographical distribution of *Shigella* infection risk to guide and inform prospective roll-out efforts towards high-priority populations.^7^

Recent advances in computing, remote sensing, and geostatistical methods, coupled with the increased availability of spatially referenced environmental data, have led to the generation of continent-wide and quasi-global high-resolution model-based maps of many infectious diseases.^8^ Although such maps have been generated for all-cause diarrhoea,^9^ so far, those specific to *Shigella* have only used indirect estimation methods. One study used estimates of overall diarrhoea-related mortality and morbidity and adjusted these by published pathogen-specific attributable fractions of diarrhoea with *Shigella* aetiology to obtain national-level incidence and death rates.^1^ Another used a similar approach, but focused on 11 African countries to derive subnational, province-level estimates based partly on covariates ascertained through household surveys.^10^ Notably, these analyses did not attempt to account for intra-annual seasonal and temporal variation in transmission, or spatial variation in disease burden below the administrative unit level. Because these analyses focused on *Shigella*-attributable morbidity and mortality, they could not account for subclinical infections. These subclinical infections are common, might contribute to transmission and overall *Shigella* prevalence, and have adverse sequalae even in the absence of overt disease.^11^

**Research in context**

**Evidence before this study**

Before undertaking this study, we searched for previously published attempts to map the burden of *Shigella* in low-income and middle-income countries since 2005 using PubMed, ResearchGate, and Google. Very few such publications existed. One study used estimates of overall diarrhoea-related mortality and morbidity and adjusted these by published pathogen-specific attributable fractions of diarrhoea with *Shigella* aetiology to arrive at national-level incidence and death rates. Another used a similar approach, but focused on 11 African countries to derive subnational, province-level estimates based partly on covariates ascertained through household surveys. Notably, these analyses did not attempt to account for intra-annual seasonal and temporal variation in transmission, or spatial variation in disease burden below the administrative unit level. Because these analyses focused on *Shigella*-attributable morbidity and mortality, they could not account for subclinical infections, which are common, might contribute to transmission and overall Shigella prevalence, and have adverse sequalae even in the absence of overt disease.

**Added value of this study**

This study is to our knowledge the first to apply an individual participant data meta-analysis approach to map *Shigella* prevalence at a subregional level and across multiple continents. It is also the first study to model the comparative effects of a set of determinants that vary on different scales, from the individual to the wider macroclimate, and, for several such determinants, include temporal variability at daily resolution. The data sources and analytical methods were selected to support spatial inference, resulting in a dataset of unparalleled scale and scope, and allowing for the first time the derevation of spatiotemporally complete, quasi-global predictions of *Shigella* infection rates as a function of climatic, environmental, and sociodemographic factors that can be extracted at specific locations. The findings could inform the design and selection of sites for forthcoming phase 3 trials of *Shigella* vaccines, and populations living in our identified hotspots could be prioritised for future vaccine roll-out campaigns. Furthermore, determining seasonal patterns of *Shigella* prevalence can be used to inform clinical diagnoses and to optimise the timing of health interventions.

**Implications of all the available evidence**

Risk of Shigella infection in low-income and middle-income countries is highly sensitive to both macro-scale and interdiurnal variation in temperature and other climatological factors, which make conditions in sub-Saharan Africa particularly propitious for propagation of the bacteria. Bands of elevated prevalence are predicted to be in the immediate north of the Congo basin and Great Lakes region, just to the north of the Tropic of Capricorn, and in tropical coastal west Africa. Zones of high predicted prevalence also occur in South America, Caribbean Central America, the Ganges–Brahmaputra Delta, and the island of New Guinea among others. These zones of high predicted prevalence might reflect the secondary influence of static environmental and household-level risk factors. Living in a household that practises open defecation (ie, that has no sanitation facility) reduces the risk of Shigella infection by a slightly larger protective effect than that of having an improved sanitation facility. This finding contradicts the widely held perception that open defecation promotes transmission of infectious intestinal diseases, and carries the important, policy-relevant implication that, from the perspective of childhood Shigella prevention, having no toilet at all is preferable to an inadequate one.

The goal of this study was to produce high-resolution maps of predicted *Shigella* prevalence across LMICs. To do this, we modelled the spatiotemporal variation in childhood infection using covariates with quasi-global coverage and an analytical approach that provides insights into drivers of *Shigella* transmission, risk, and seasonality. A specific guiding hypothesis was that the probability of *Shigella* infection in a given location varies day to day as a function of antecedent weather conditions, particularly temperature, for children experiencing diarrhoeal episodes.^5^, ^12^

## Methods

### Objective and scope

The objective of this analysis was to estimate the percent prevalence of enteric *Shigella* infection in three separate age groups (0–11 months, 12–23 months, and 24–59 months) and three syndrome strata (asymptomatic, uncomplicated [community-detected] diarrhoea, and medically attended diarrhoea) at all locations throughout the world's LMICs (as defined in the appendix p 2).

Ethics approval for this study was given by the University of Virginia Institutional Review Board for Health Sciences Research (IRB-HSR 21544) and the Johns Hopkins University Homewood Institutional Review Board (study number HIRB0011882). Each contributing study obtained ethics approval from their respective institutions, and written informed consent was obtained from participants’ caregivers for collection, storage, and analysis of biological specimens as detailed elsewhere. All contributing authors and investigators gave their consent to analyse and publish the data.

### Data sources and outcome variable

To compile a dataset representative of diverse geographical and climatic contexts, data were sourced and compiled from multiple completed studies identified through non-systematic, exploratory literature review and professional networks according to inclusion criteria and within an individual participant data meta-analysis (IPD-MA) framework described previously and in the appendix (p 2).^5^ Investigators on eligible studies were contacted with a request to access data from individual participants and, if they agreed, data use agreements were established with the collaborating institution. The outcome of interest was *Shigella* infection status, which was ascertained by PCR performed on diarrhoeal and surveillance (asymptomatic) stool samples collected from children aged 59 months or younger and was treated as a binary variable (positive or negative). The *Shigella* stool positivity rate (the probability of PCR-detection) was modelled as an approximation of the prevalence of paediatric *Shigella* infection. Discrete infection episodes were defined as previously described.^5^

### Covariates

#### Study design and symptom status

Data came from studies that varied in size, scope, and design. Health-facility-based case–control and surveillance studies actively recruited patients seeking care for gastrointestinal syndromes ranging in severity from uncomplicated to acute, often watery, diarrhoea and dysentery (bloody diarrhoea). By design, case–control studies of this kind—such as the Global Enteric Multicenter Study (GEMS),^13^ which took place in sites in seven countries—collected equal numbers of samples from asymptomatic controls as from diarrhoea cases. Health-facility-based surveillance studies—such as the Delivery of Oral Cholera Vaccine Effectively (DOVE) study in Cameroon^14^ and the Diarrhoeal Sentinel Surveillance Programme (DSSP) in South Africa^15^—only recruited on the basis of diarrhoea status and therefore did not include asymptomatic samples.

Community-based cohort, surveillance, and case–control studies recruited participants based on the communities in which they live. Cohort studies collected stool samples according to predefined schedules, and therefore purported to collect symptomatic, diarrhoeal samples at roughly the rate at which the syndrome occurs in the community, although a majority of samples are obtained while the participants are not experiencing gastrointestinal symptoms. Notable among these studies was the eight-country Malnutrition and Enteric Disease (MAL-ED) cohort study.^16^ Just one study (RECODISA) had a community-based case–control design,^17^ actively recruiting diarrhoea cases occurring in the community and matching them with asymptomatic controls. By design, cases in that study had less severe diarrhoea than their equivalents in health-facility based studies.

Intervention trials could be health facility based—such as the placebo-controlled Rotavac vaccine efficacy trial in sites in three Indian cities^18^—or community based—such as the Sanitation Hygiene Infant Nutrition Efficacy (SHINE - Zimbabwe)^19^ and WASH HELPS (Laos)^20^ trials of water, sanitation, and hygiene interventions.

13 (65%) of the included studies had health-facility-based designs. Therefore, results for diarrhoeal samples from symptomatic individuals were over-represented in the overall database relative to the frequency of diarrhoea occurrence in the general population despite making up just a quarter of the total samples. This overestimation could lead to overestimation of risk for *Shigella*, a pathogen strongly associated with diarrhoea and dysentery. To adjust for this limitation, a categorical variable was included indicating whether the stool sample was collected while the child was asymptomatic or symptomatic (experiencing a diarrhoeal episode of any severity) and among the symptomatic samples, whether it was from a study with a community-surveillance or health-facility-based design. This adjustment was to account for the assumed differential pathogen positivity rates in symptomatic samples and, especially, diarrhoea for which facility care was sought.^5^ The inclusion of this three-category term allowed the predicted stool *Shigella*-positivity rate to be modelled separately for three symptom status categories: (1) asymptomatic, (2) community-detected diarrhoea, and (3) medically attended diarrhoea (cases identified in patients from a health facility). Samples collected from community cohort participants while asymptomatic were treated as equivalent to those from control cases in case–control studies.

#### Age

Participants’ ages at sample collection were grouped into three age strata (0–11 months, 12–23 months, and 24–59 months) to adjust for the well documented age-dependent risk of childhood *Shigella* and to predict risk separately for age groups commonly reported in studies of enteropathogen burden. Age, symptom status, and study design are hereafter referred to collectively as the control variables, because they were included in the model to control for confounding and are the only variables for which separate predictions were made for each value, rather than letting their values vary spatially.

#### Participant-level and household-level covariates

Most contributing studies conducted baseline and follow-up assessments of risk and vulnerability of *Shigella* transmission. These data (summarised in the appendix pp 12–13), were recoded to match as closely as possible to standardly used definitions of variables and, where missing or not collected by some studies, imputed or interpolated from household survey data according to methods described previously and in the appendix (p 7).^3^

#### Environmental spatial covariates

A suite of time-static environmental and sociodemographic spatial covariates (summarised in the appendix p 10) available in raster format were compiled based on their hypothesised or demonstrated associations with diarrhoeal disease outcomes.^9^ Variable values were extracted at each child's georeferenced location according to methods described in the appendix (p 14).

#### Time-varying hydrometeorological variables

We selected a set of historical daily estimates of hydrometeorological variables derived from Earth Observation and model-based re-analysis (appendix p 11). This selection was based on their demonstrated or hypothesised potential to influence enteric pathogen transmission, extracted from the Global Land Data Assimilation System (GLDAS;^21^ version 2.1), where appropriate, standardised to local distributions and summarised over a lagged period of exposure, by use of methods described previously and in the appendix (p 14).^5^

### Statistical analysis

For the model fitting, generalised multivariable models were fitted to the binary outcome of infection status to estimate predictive probabilities of positivity for *Shigella* infection using the additive^22^ R packages (a wrapper function for *mgcv*^23^ developed for this analysis and extended as a general-purpose software tool). An initial generalised linear model (GLM) including only the control variables was fitted to the outcome to serve as a reference for intercomparisons with subsequent models of increasing complexity in a repeated *k*-fold cross-validation analysis, namely: a GLM that included all non-hydrometeorological variables modelled as linear; a GLM that included all variables modelled as linear; and a final generalised additive model (GAM) with splines specified for the hydrometeorological variables to allow for the previously documented non-linearity of their associations with *Shigella.*^5^ The performance of the models was compared on the basis of several in-sample and out-of-sample classification metrics by use of cross-validation (appendix p 20), and the importance of variables was identified for all categories using accumulated local effects (ALE)^24^ and the average expected marginal contribution (Shapley values; appendix p 20).^25^ An interaction between air temperature and symptom status was specified, based on a hypothesis and evidence from exploratory analyses (appendix p 19) that temperature differentially affects the probability of diarrhoeal samples being *Shigella* positive compared with samples from asymptomatic individuals. Similarly, an interaction between precipitation and soil moisture was specified based on the hypothesis that associations with rainfall on diarrhoea-causing pathogens might be subject to effect modification by antecedent wetness conditions, such that the impact of extreme rainfall is increased following drier conditions.^26^

The final model results were used to make separate predictions for each syndrome and age stratum by use of the coefficient estimates for the corresponding control variables at each combination of their values. Raster files of the values of each other covariate across the geographical extent of the domain of interest, LMICs, were obtained or generated as described in the appendix (p 15). For the binary household-level and participant-level covariates, coefficient estimates of the probability of *Shigella* infection in the comparison category relative to the reference group were adjusted by the geographically varying proportion coverage or prevalence stored in raster format. Seasonality was explored by extracting and plotting time series of daily predictions at six key illustrative locations; these were settlements in areas of high and low prevalence in LMICs in each of the three regions—Africa, Asia, and the Americas.

A threshold of 5% (two-tailed) was set for reporting statistical significance of associations. Analyses were carried out using Stata (version 16),^27^ R (version 4.0.3),^28^ and ArcMap (version 10.8),^29^ and PRISMA-IPD^30^ and GATHER^31^ guidelines were followed (appendix pp 22–27).

## Results

**Figure 1:**
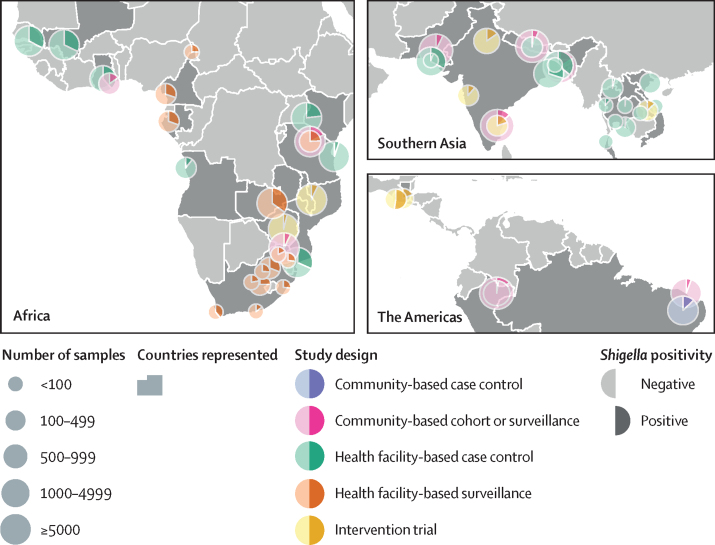
Locations of the sites and designs of the studies contributing data to this analysis, and number of samples included in the analysis and *Shigella*-positivity rates from each site

20 studies contributed data from 23 countries with a range of latitudes spanning the tropics and subtropics, including locations in Central America, South America, sub-Saharan Africa and south and southeast Asia, and spanning overlapping follow-up periods from 2007 to 2018. Figure 1 shows the locations, number of samples, and the proportion of positive samples at each study site, and the appendix (pp 3–6) summarises key features of contributing studies.

The table shows the distribution of stool samples in the pooled database with available positivity status for *Shigella* spp included in this analysis by age group and child's symptom status. Almost three-quarters of diagnostic results (74·0%) were from surveillance samples, collected from asymptomatic individuals, while of the remaining diarrhoeal samples, almost twice as many were collected from children presenting at health facilities as from those recruited in communities (16·9% *vs* 9·2%). Roughly equal numbers of samples were collected from children in the first and the second years of life (46·6% *v*s 44·0% of the database), while those from older children (aged ≥2 years) made up under 10% of the database.

**Table:** Number (and percent) of stool samples with available *Shigella* spp positivity status used in this analysis by child's age and diarrhoea symptom status

	**Asymptomatic**	**Diarrhoeal symptoms**		**Total**
		Community-detected	Medically attended	
0–11 months	23 013 (34·6%)	3190 (4·8%)	4796 (7·2%)	30 999 (46·6%)
12–23 months	22 753 (34·2%)	2577 (3·9%)	3945 (6·0%)	29 275 (44·0%)
24–59 months	3514 (5·3%)	328 (0·5%)	2447 (3·7%)	6289 (9·4%)
Total	49 280 (74·0%)	6095 (9·2%)	11 188 (16·9%)	66 563 (100%)

Community-detected diarrhoea refers to diarrhoea cases identified in community-based surveillance. Medically attended diarrhoea refers to diarrhoea cases identified in health facility-based studies.

The appendix (p 20) summarises performance statistics of the interim and final models and two metrics of importance for each variable included in the final model—Shapley values and ALE—which are ordered along the y-axis by the average of the two. By every metric, the performance of the models improved with increasing complexity, and the final GAM that modelled the hydrometeorological variables as non-linear showed improved performance over the GLMs that treated them as linear. Child's age was the most important contribution by both metrics, followed by the other control variables (symptom status or study design). The next four most important factors were all time-varying hydrometeorological variables (temperature, wind speed, relative humidity, and soil moisture), followed by three static environmental variables (irrigated areas, cropland areas, and enhanced vegetation index). The most important household-level variable was presence of an improved sanitation facility, which ranked 12th, while the most important subject-level variable, stunting, ranked far down the list at 22nd.

**Figure 2:**
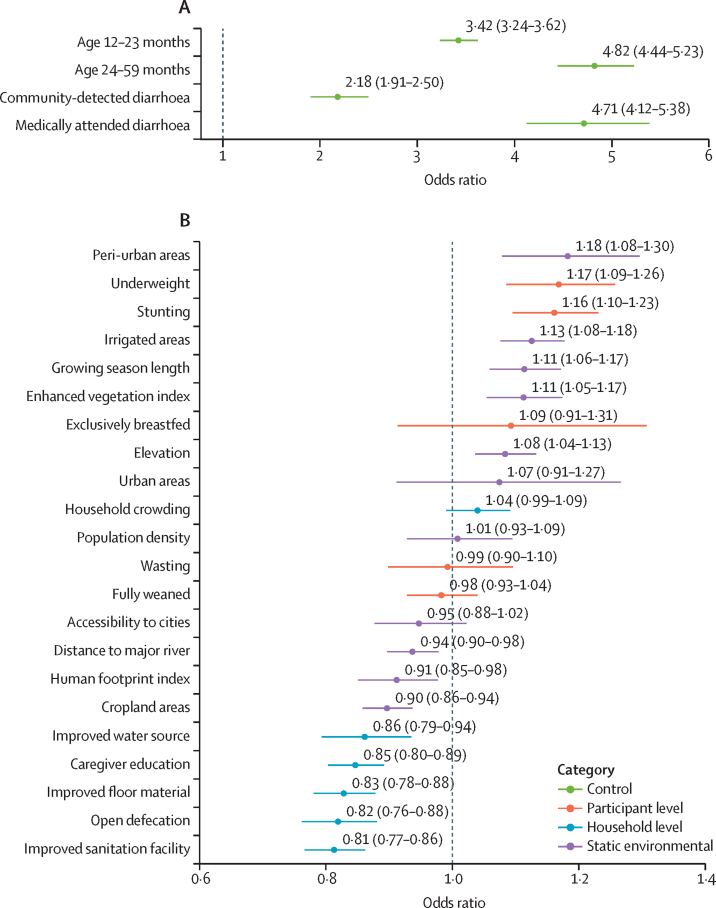
Odds ratios of *Shigella* infection predicted by the conditional effects of subject-level and household-level variables in the final model The reference category for age is the 0–11 month group and the coefficients for the diarrhoea categories are in comparison with asymptomatic children from community surveillance studies. Odds ratios for continuous static environmental variables are for one standard deviation increase.

**Figure 3:**
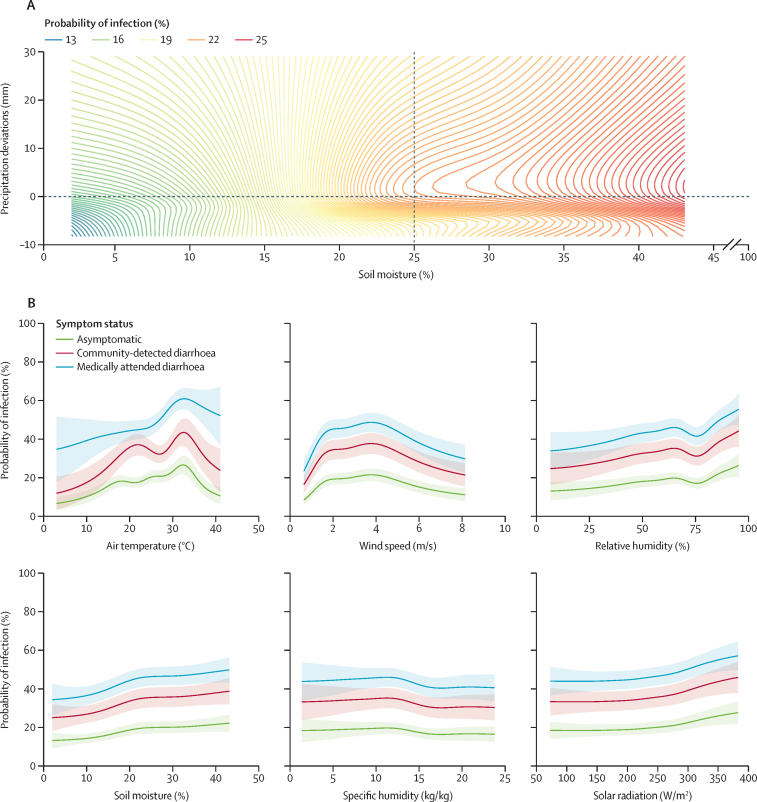
Probabilities of *Shigella* infection predicted by the conditional effects of time-varying hydrometeorological variables in the final model Precipitation deviations are relative to long term (2005–19) site-specific averages.

Figures 2 and 3 show the probability of *Shigella* infection predicted by the conditional effects of the most important hydrometeorological variables (and their interactions) in the final model (figure 3) and the odds ratios (ORs) for the other variables (figure 2). Children aged 24–59 months had higher odds of *Shigella* positivity (OR=4·82 [95% CI 4·43–5·21]) than those younger than 1 year, as did those aged 12–23 months (OR=3·42 [3·24–3·61]; figure 2). Temperature exhibited a non-linear, asymmetrical, inverse U-shaped association with the probability of *Shigella* infection, and was most marked for symptomatic children (figure 3). Probability increased steadily across the first three quartiles of the temperature distribution, peaking at a value of around 34°C with a 43% probability of *Shigella* detection for uncomplicated diarrhoea cases and 26% for asymptomatic children, before decreasing above that threshold. An interaction was observed between precipitation and soil moisture, such that probability of positivity was low across the entire range of the precipitation distribution during very dry soil conditions, and moderate during periods of below-average rainfall, but when both variables were above average concurrently, the probability of *Shigella* detection increased and exceeded 20%. Wind speed also had an asymmetrical, inverse U-shaped association with probability of *Shigella* infection but skewed to the other side, with the peak risk occurring at speeds of 4 m/s. Relative humidity and solar radiation had a broadly direct effect on risk of *Shigella* infection, and specific humidity had a low-magnitude inverse association with the probability of *Shigella* infection.

Living in peri-urban areas had the largest direct association with *Shigella* infection of the non-hydrometeorological variables (OR=1·18 [95% CI 1·07–1·29]) compared with rural residents, although the equivalent estimate for fully urban areas was non-significant. Two anthropometric markers were next, with moderate-to-severely underweight children or children with stunting showing strong evidence of increased odds of testing positive for *Shigella* (OR=1·17 [1·08–1·26] for underweight and OR=1·16 [1·09–1·23] for stunting). The other participant-level variables—exclusive breastfeeding, full weaning, and wasting—had negligible, non-significant effects. The largest inverse associations among the non-hydrometeorological variables were for five of the household-level covariates. Living in a household that has an improved sanitation facility or practises open defecation decreased the odds of *Shigella* detection (OR=0·81 [0·76–0·86] for improved sanitation facility and OR=0·82 [0·76–0·88] for open defecation) compared with an unimproved facility; having improved floor material (OR=0·83 [0·78–0·88]), a caregiver who completed primary education (OR=0·85 [0·81–0·89]), or an improved water source (OR=0·86 [0·79–0·93]) had slightly smaller protective effects. Of the remaining environmental variables, strong evidence was found that irrigated areas (OR=1·13 [1·08–1·18]), growing season length (OR=1·11 [1·05–1·17]), enhanced vegetation index (OR=1·11 [1·05–1·17]), and elevation (OR=1·08 [1·03–1·13]) were associated with higher risk of *Shigella* infection, whereas cropland areas (OR=0·90 [0·86–0·94]), the human footprint index (OR=0·91 [0·85–0·97]), and distance to a major river (OR=0·94 [0·90–0·98]), were associated with a lower risk.

**Figure 4:**
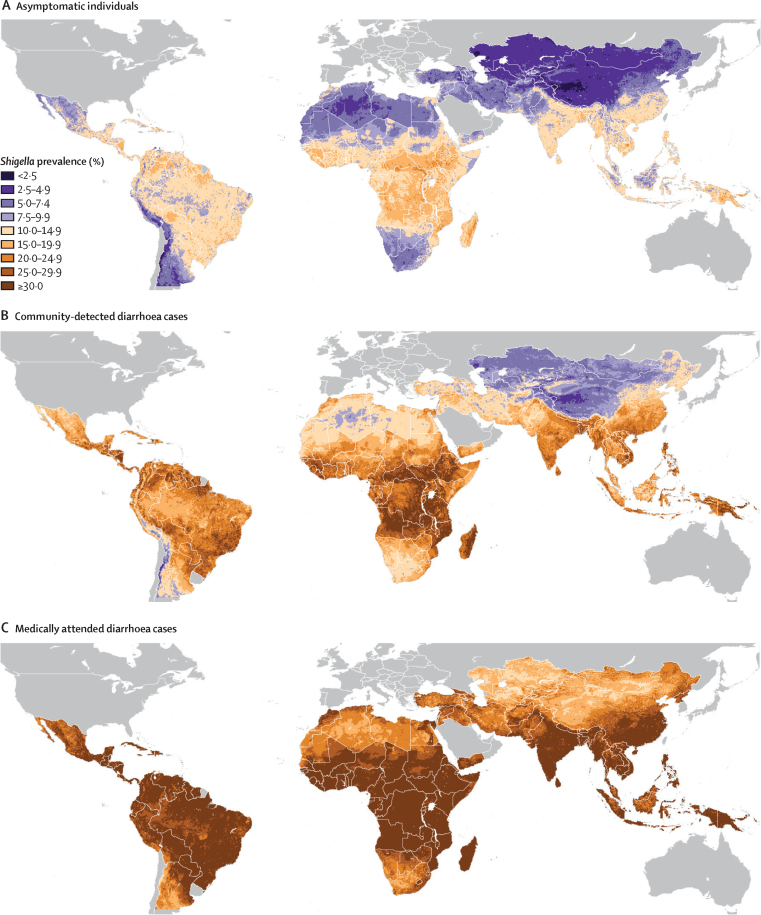
Geographical distribution of the annual mean prevalence of *Shigella* infection in children aged 12–23 months predicted by the final model by symptom status

Figure 4 shows the geographical distribution of the annual mean prevalence of *Shigella* positivity predicted by the model for children aged 12–23 months in 2018 for each of the three symptom strata. Predicted prevalence of *Shigella* in asymptomatic individuals (figure 4) varied from below 2·5% in areas of western China, central Asia, and the Argentinian Andes to over 20% in small pockets of Central America (notably eastern Nicaragua), northern South America, tropical sub-Saharan Africa, Bangladesh, southeast Asia (notably northeastern Cambodia), and Papua New Guinea. For individuals with uncomplicated diarrhoea (figure 4), the distribution was shifted upward such that there were large areas with predicted prevalence of higher than 25%, including almost all the territories of the Central African Republic and South Sudan. Large areas of high prevalence were also predicted across the other countries around the African Great Lakes, coastal West Africa, Angola, Madagascar, and Bangladesh, with more delimited pockets discernible in Caribbean Central America, the interiors of Bolivia, Colombia, and Venezuela, and continental southeast Asia. The predicted prevalence in individuals with medically attended diarrhoea (figure 4) exceeded 30% over almost the entirety of the tropics (with some exceptions such as parts of Borneo and the Andes) as well as large areas of subtropical Mexico, eastern China, and northeastern Argentina. Across all three symptom strata, the lowest risk was predicted over the greater Central Asia region (including Mongolia and western China), Saharan North Africa, the Andes, and southern Africa.

**Figure 5:**
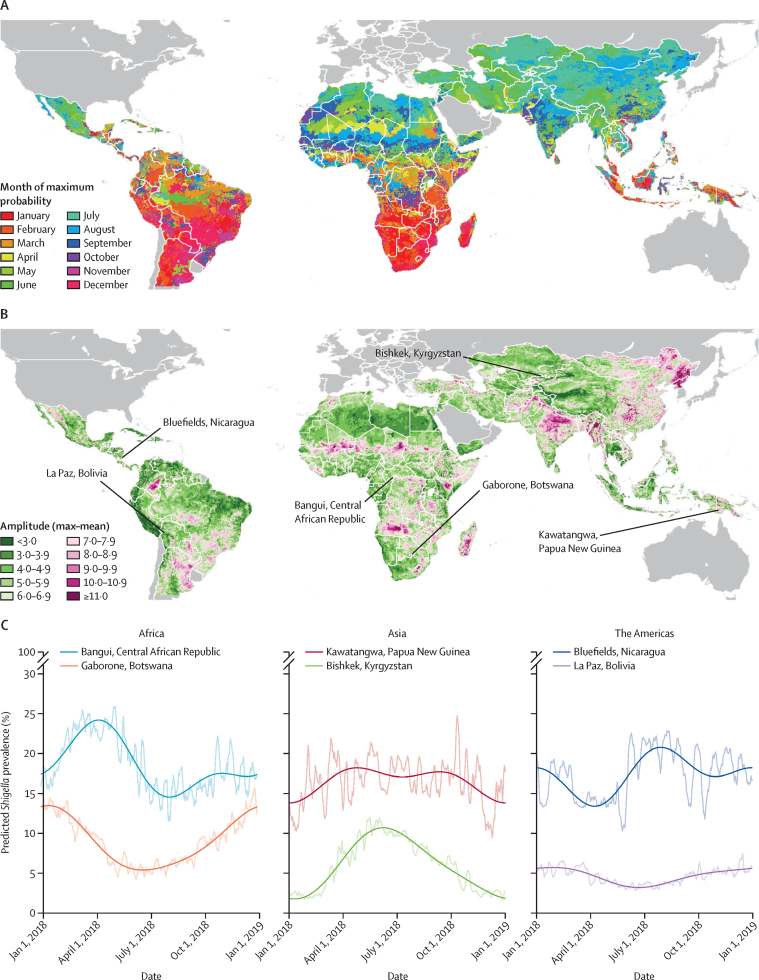
Seasonality metrics for *Shigella* positivity in asymptomatic children aged 12–23 months predicted by the model: (A) Timing of annual peak (month in which maximum daily predicted positivity in 2018 occurs); (B) Amplitude of annual peak (percentage point difference between the maximum and mean daily values in 2018); (C) Time series plots of the daily (transparent) and smoothed (opaque) estimates of *Shigella* prevalence in asymptomatic children aged 12–23 months over 2018 at six illustrative high and lower prevalence locations in Africa, Asia, and the Americas. Smoothing was done by regression with annual and biannual harmonic terms.

The supplementary animation (video) shows the temporal variation in the predictions for each day of the year 2018, while figure 5 shows the geographical distribution of two seasonality parameters: the timing (month in which the maximum prevalence was predicted), and amplitude (difference between maximum and minimum predicted prevalence) of the annual seasonal peak, as well as the annual time series of daily and smoothed predictions for asymptomatic children aged 12–23 months over the same year at six illustrative locations. Patterns of seasonality varied from one location to the next. The high prevalence in the Central African Republic location showed a boreal spring peak in early April, compared with the low prevalence in the Botswana location where a December-to-February peak was evident. The high prevalence in the Papua New Guinea location showed low amplitude, with only slightly higher values in the March–October period, while in Bishkek, Kyrgyzstan, a more marked mid-year peak of 11% was observed, subsiding to very low prevalence at the beginning and end of year. A mid-year and secondary end-of-year peak was discernible in high-prevalence Bluefields, Nicaragua, in contrast to La Paz, Bolivia, where year-round low prevalence showed little seasonal variation.

## Discussion

This IPD-MA and Bayesian GAM analysis revealed not only small-scale zones of potential elevated transmission risk in LMICs, but also generalisable evidence about the relative influence of different drivers of *Shigella* transmission. Notable among these findings is that the risk of *Shigella* infection increases with warmer conditions, especially in children exhibiting and seeking care for diarrhoeal symptoms, but only up to a point in the low-to-mid 30s °C. Soil saturation levels and rainfall volume also interact to determine risk, while children living in peri-urban areas and with moderate-to-severe stunting and underweight are particularly vulnerable. This increased risk makes conditions in sub-Saharan Africa particularly propitious for propagation of the bacterium. Bands of elevated prevalence were predicted to the immediate north of the Congo basin and Great Lakes region, just to the north of the Tropic of Capricorn, and in tropical coastal West Africa, consistent with the high rates reported in studies not included in this analysis, such as in the Central African Republic,^32^ Guinea Bissau,^33^ Mozambique,^34^ and Madagascar.^35^ Zones of high predicted prevalence also occurred in South America, Caribbean Central America, the Ganges–Brahmaputra Delta, and the island of New Guinea among others, and might reflect the secondary influence of static environmental and household-level risk factors. Furthermore, the seasonal variability in infection risk within the same locations revealed by these findings could be relevant for diagnosis and treatment and for optimal timing of health system interventions.

The results confirm that the risk of *Shigella* infection has a strong association with age—the highest prevalence occurring in older children (24–59 months)—and diarrhoea symptom status, consistent with effects documented previously.^2^ While these control variables ranked highest in importance and had the largest effect sizes, they were closely followed by hydrometeorological variables including temperature, wind speed, relative humidity, and soil moisture. These associations exhibited considerable non-linearity and were consistent in shape, although larger in magnitude, than those identified from an earlier version of this dataset.^5^ Atmospheric and hydrological factors probably affect the survival and dispersal of bacteria such as *Shigella* outside the host, where warm and moist conditions might prolong their viability.^5^, ^36^ The importance of temperature in *Shigella* transmission also confirms previously reported findings. A meta-analysis found a relative risk (RR) of *Shigella* diagnosis of 1·07 for each 1°C increase in temperature,^4^ while the equivalent for a 5°C increase in data pooled from several hundred sentinel sites across China was 1·31.^37^ However, we also demonstrated that *Shigella* prevalence dropped sharply in the upper temperature extreme above a value of around 33°C, and that the inverse U-shape of this association was more pronounced for symptomatic than asymptomatic *Shigella*.

Although some studies have found no association between rainfall and *Shigella* infection or shigellosis,^37^ here we observed a direct association between precipitation and *Shigella* infection, as well as an interaction with soil moisture. *Shigella* prevalence was highest when these environmental factors were above their average values. Kraay and colleagues hypothesise that extreme rainfall increases diarrhoea incidence to a greater extent if it follows a drier period;^26^ however, these findings suggest that such an effect, if true, is not mediated by *Shigella*. This compounding of soil saturation conditions on precipitation is, however, consistent with findings from an interrupted time-series analysis of a large flood related to the phenomenon La Niña in the study site in Loreto, Peru, where an increase in *Shigella* infection was observed, but only in the later period of the flood.^6^

Although household factors ranked lower in importance than environmental and control variables, several significant protective associations of these variables were observed. Living in a household that practises open defecation (ie, has no sanitation facility) conferred lower odds of *Shigella* infection by almost a fifth (18%)—a protective association almost equal to that of having an improved sanitation facility (19%). This finding goes against the widely held perception that open defecation promotes transmission of infectious intestinal diseases,^38^ and carries the implication that, from the perspective of childhood *Shigella* prevention, having no toilet at all is preferable to an inadequate one (although open defecation might still be a risk factor for other diarrhoeal pathogens and adverse health and social outcomes).^39^ Furthermore, these effect sizes are smaller than those from a trial of pour-flush toilets with septic tanks in Maputo, Mozambique, which saw prevalence of *Shigella* in children younger than 2 years reduce by half.^34^ While the further protective effects of caregiver primary education and covered floors (both ~15%) are consistent with those in our previous study of a smaller version of this dataset,^3^ the 14% reduced odds of *Shigella* infection conferred by having an improved water source is modest compared with findings from Manhiça District Mozambique showing that water availability is among the most protective factors against *Shigella* infection in children younger than 2 years.^40^

The direct association of two indicators of undernutrition—moderate-to-severe underweight and stunting—with *Shigella* infection were of similar magnitude to the protective effects of household factors, meaning that children with a weight-for-age or height-for-age *Z*-score 2 or more standard deviations below that of a healthy reference population were at increased risk of *Shigella* infection. This is further evidence of the well documented bidirectional interplay between malnutrition and enteric infections, and consistent with findings from multisite studies that both symptomatic^41^ and asymptomatic^42^ untreated *Shigella* infections in young children are associated with later adverse anthropometric outcomes. No evidence was found for feeding status being associated with positivity for *Shigella* infection, which is notable because elsewhere early introduction of complementary foods was associated with a 10% increased RR of infection with *Shigella*.^2^ However, it is consistent with a comparative analysis of the effect of full breastfeeding on multiple enteropathogens, which found associations with some viruses and bacteria, but no evidence of an association with *Shigella*.^43^

This study had various limitations. Merging data from multiple separate studies introduces potential error and bias. To minimise this limitation, we restricted the outcome data to *Shigella* status ascertained by PCR—a diagnostic method that is highly sensitive and specific across all settings, and by standardising the sources and methods for extracting, imputing, or otherwise processing the covariate data so that they are comparable across all sites. We also adjusted for the fixed effects of study design (health facility based versus community based) as strata within the diarrhoeal syndrome category. A further limitation relating to this last point is our implicit assumption about how *Shigella* prevalence differs between symptomatic and asymptomatic individuals and as an artifact of differences in study design between health-facility-based and community-based surveillance. We included the far more numerous asymptomatic samples in the analysis in part under the assumption that they contribute information about overall transmission risk—ie, that conditions that promote asymptomatic *Shigella* carriage also determine the probability of positivity in cases of both uncomplicated and more severe diarrhoea. Although the inclusion of an interaction term allowed this association to vary by temperature rather than being merely additive, in reality, the differential between community and facility-based observations is likely to be mediated by a multiplicity of factors, including care-seeking behaviour (itself a function of socioeconomic status, education, and health system accessibility and infrastructure) and geographical location. A thorough exploration of the factors that determine care-seeking behaviour for diarrhoea was beyond the scope of this analysis and would require information that might not be available for all included studies, although it should be the subject of future research. Instead, we based our model on a small number of a priori assumptions that were parsimonious and prespecified. Furthermore, the potential for bias might be exacerbated by imbalances in the data, not just the disproportionate representation of asymptomatic sample, but also the tendency for studies of different designs to be located in different regions. For example, studies from South America tended to be community based whereas those from southern Africa were predominantly health facility based. This finding reflects the hitherto piecemeal nature of global enteropathogen surveillance and the need for protocols that are standardised across geographical regions, which is beginning to be addressed by initiatives such as the Global Pediatric Diarrhea Surveillance Network.^44^ Relatedly, it was only possible to directly estimate *Shigella* PCR-positivity in diarrhoeal samples and not the *Shigella*-specific attributable burden of diarrhoea, which, because of the pervasiveness of co-infection with multiple diarrhoea-causing agents, are far from equivalent (the former tending to overestimate the latter considerably). To address this limitation, the prediction estimates for children with diarrhoea provided in supplementary files can be adjusted downward using generalisable or context-specific *Shigella*-attributable fractions as estimates of these values become available. Similarly, these prevalence estimates should not be conflated with incidence, which could be higher in settings where repeated diarrhoeal episodes are common even where point prevalence is low.

In conclusion, the spatiotemporal distribution of *Shigella* risk in LMICs appears sensitive to both macro-scale and interdiurnal variation in temperature and other climatological factors. These findings should inform the design and selection of sites for forthcoming phase 3 *Shigella* vaccine trials,^7^ and populations living in these hotspots should be considered high priority for eventual vaccine roll-out campaigns.

## Data sharing

The epidemiological data used in this analysis contain identifiable human subject data, which cannot be disseminated under the terms of the IRB and data use agreements with contributing institutions. Investigators from contributing studies can be contacted with reasonable request for data access. Data from the MAL-ED and GEMS studies are available from the ClinEpiDB website. GLDAS data are disseminated as part of the mission of NASA's Earth Science Division and archived and distributed by the Goddard Earth Sciences (GES) Data and Information Services Center (DISC). Other input data are available from the sources cited in the appendix. Processed versions in raster/TIFF format are available upon reasonable request to the corresponding author as is statistical source code. Output model predictions of probabilities and standard errors are available for each of nine age groups/symptom stratum combinations at the following GitHub repository https://github.com/joshcolston/Badr_Shigella_predictions.

## Declaration of interests

JG and MM report grants from PanTheryx, during the conduct of the study; NP reports grants from GlaxoSmithKline, during the conduct of the study; the remaining authors declare no competing interests.

## Supplementary Materials

Supplementary appendix

Supplementary video Shigella seasonality in low-income and middle-income countries Animated time series of daily Shigella prevalence estimates in asymptomatic children aged 12–23 months, Jan 1–Dec 31, 2018 **YouTube url:**https://youtu.be/fdiA5_XrEDM

## References

[1] Khalil IA, Troeger C, Blacker BF (2018). Morbidity and mortality due to shigella and enterotoxigenic Escherichia coli diarrhoea: the Global Burden of Disease Study 1990–2016. Lancet Infect Dis.

[2] Rogawski Mcquade ET, Shaheen F, Kabir F (2020). Epidemiology of Shigella infections and diarrhea in the first two years of life using culture-independent diagnostics in 8 low-resource settings. PLoS Negl Trop Dis.

[3] Colston JM, Faruque ASG, Hossain MJ (2020). Associations between household-level exposures and all-cause diarrhea and pathogen-specific enteric infections in children enrolled in five sentinel surveillance studies. Int J Environ Res Public Health.

[4] Chua PLC, Ng CFS, Tobias A, Seposo XT, Hashizume M (2022). Associations between ambient temperature and enteric infections by pathogen: a systematic review and meta-analysis. Lancet Planet Health.

[5] Colston JM, Zaitchik BF, Badr HS (2022). Associations between eight earth observation-derived climate variables and enteropathogen infection: an independent participant data meta-analysis of surveillance studies with broad spectrum nucleic acid diagnostics. Geohealth.

[6] Colston J, Paredes Olortegui M, Zaitchik B (2020). Pathogen-specific impacts of the 2011–2012 La Niña-associated floods on enteric infections in the MAL-ED Peru Cohort: a comparative interrupted time series analysis. Int J Environ Res Public Health.

[7] Pavlinac PB, Rogawski McQuade ET, Platts-Mills JA (2022). Pivotal Shigella vaccine efficacy trials-study design considerations from a Shigella Vaccine Trial Design Working Group. Vaccines (Basel).

[8] Chowell G, Rothenberg R (2018). Spatial infectious disease epidemiology: on the cusp. BMC Medicine.

[9] Reiner RC, Wiens KE, Deshpande A (2020). Mapping geographical inequalities in childhood diarrhoeal morbidity and mortality in low-income and middle-income countries, 2000–17: analysis for the Global Burden of Disease Study 2017. Lancet.

[10] Bagamian KH, Anderson JD, Muhib F (2019). Heterogeneity in enterotoxigenic *Escherichia coli* and shigella infections in children under 5 years of age from 11 African countries: a subnational approach quantifying risk, mortality, morbidity, and stunting. Lancet Glob Health.

[11] McQuade ETR, Scharf RJ, Svensen E (2022). Impact of shigella infections and inflammation early in life on child growth and school-aged cognitive outcomes: findings from three birth cohorts over eight years. PLoS Negl Trop Dis.

[12] Gao L, Zhang Y, Ding G (2014). Meteorological variables and bacillary dysentery cases in Changsha City, China. Am J Trop Med Hyg.

[13] Kotloff KL, Blackwelder WC, Nasrin D (2012). The Global Enteric Multicenter Study (GEMS) of diarrheal disease in infants and young children in developing countries: epidemiologic and clinical methods of the case/control study. Clin Infect Dis.

[14] Debes AK, Xiao S, Liu J (2021). Characterization of enteric disease in children by use of a low-cost specimen preservation method. J Clin Microbiol.

[15] Page NA, Seheri LM, Groome MJ (2018). Temporal association of rotavirus vaccination and genotype circulation in South Africa: observations from 2002 to 2014. Vaccine.

[16] MAL-ED Network Investigators (2014). The MAL-ED study: a multinational and multidisciplinary approach to understand the relationship between enteric pathogens, malnutrition, gut physiology, physical growth, cognitive development, and immune responses in infants and children up to 2 years of. Clin Infect Dis.

[17] Lima AAM, Oliveira DB, Quetz JS (2019). Etiology and severity of diarrheal diseases in infants at the semiarid region of Brazil: a case-control study. PLoS Negl Trop Dis.

[18] Bhandari N, Rongsen-Chandola T, Bavdekar A (2014). Efficacy of a monovalent human-bovine (116E) rotavirus vaccine in Indian infants: a randomised, double-blind, placebo-controlled trial. Lancet.

[19] Humphrey JH, Mbuya MNNN, Ntozini R (2019). Independent and combined effects of improved water, sanitation, and hygiene, and improved complementary feeding, on child stunting and anaemia in rural Zimbabwe: a cluster-randomised trial. Lancet Glob Health.

[20] Chard AN, Baker KK, Tsai K (2019). Associations between soil-transmitted helminthiasis and viral, bacterial, and protozoal enteroinfections: a cross-sectional study in rural Laos. Parasites & Vectors.

[21] Rodell M, Houser PR, Jambor U (2004). The Global Land Data Assimilation System. Bull Am Meteor Soc.

[22] Badr HS (2021). Bindings for Generalized Additive Models (GAM). https://hsbadr.github.io/additive/.

[23] Wood SN (2011). Fast stable restricted maximum likelihood and marginal likelihood estimation of semiparametric generalized linear models. J Royal Stat Soc Series B (Stat Methodol).

[24] Apley DW, Zhu J (2020). Visualizing the effects of predictor variables in black box supervised learning models. J Royal Stat Soc Series B.

[25] Štrumbelj E, Kononenko I (2014). Explaining prediction models and individual predictions with feature contributions. Knowl Inf Syst.

[26] Kraay ANM, Man O, Levy MC, Levy K, Ionides E, Eisenberg JNS (2020). Understanding the impact of rainfall on diarrhea: testing the concentration-dilution hypothesis using a systematic review and meta-analysis. Environ Health Perspect.

[27] StataCorp. Stata Statistical Software: release 16. 2019.

[28] R Core Team. R: a language and environment for statistical computing. 2020.

[29] ESRI. ArcGIS Desktop: release 10.8. 2019.

[30] Stewart LA, Clarke M, Rovers M (2015). Preferred reporting items for systematic review and meta-analyses of individual participant data: the PRISMA-IPD Statement. JAMA.

[31] Stevens GA, Alkema L, Black RE (2016). Guidelines for accurate and transparent health estimates reporting: the GATHER statement. Lancet.

[32] Breurec S, Vanel N, Bata P (2016). Etiology and epidemiology of diarrhea in hospitalized children from low income country: a matched case-control study in Central African Republic. PLoS Negl Trop Dis.

[33] Mero S, Timonen S, Lääveri T (2021). Prevalence of diarrhoeal pathogens among children under five years of age with and without diarrhoea in Guinea-Bissau. PLoS Negl Trop Dis.

[34] Knee J, Sumner T, Adriano Z (2021). Effects of an urban sanitation intervention on childhood enteric infection and diarrhea in Maputo, Mozambique: a controlled before-and-after trial. eLife.

[35] Collard J-M, Andrianonimiadana L, Habib A (2022). High prevalence of small intestine bacteria overgrowth and asymptomatic carriage of enteric pathogens in stunted children in Antananarivo, Madagascar. PLoS Negl Trop Dis.

[36] Grinberg M, Orevi T, Steinberg S, Kashtan N (2019). Bacterial survival in microscopic surface wetness. eLife.

[37] Wang L-P, Zhou S-X, Wang X (2021). Etiological, epidemiological, and clinical features of acute diarrhea in China. Nat Commun.

[38] Mara D (2017). The elimination of open defecation and its adverse health effects: a moral imperative for governments and development professionals. J Water Sanit Hyg Dev.

[39] Saleem M, Burdett T, Heaslip V (2019). Health and social impacts of open defecation on women: a systematic review. BMC Public Health.

[40] Vubil D, Acácio S, Quintò L (2018). Clinical features, risk factors, and impact of antibiotic treatment of diarrhea caused by *Shigella* in children less than 5 years in Manhiça district, rural Mozambique. Infect Drug Resist.

[41] Nasrin D, Blackwelder WC, Sommerfelt H (2021). Pathogens associated with linear growth faltering in children with diarrhea and impact of antibiotic treatment: the Global Enteric Multicenter Study. J Infect Dis.

[42] Nasrin S, Haque MA, Palit P (2022). Incidence of asymptomatic shigella infection and association with the composite index of anthropometric failure among children aged 1–24 months in low-resource settings. Life.

[43] McCormick BJJ, Richard SA, Murray-Kolb LE (2022). Full breastfeeding protection against common enteric bacteria and viruses: results from the MAL-ED cohort study. Am J Clin Nutr.

[44] Cohen AL, Platts-Mills JA, Nakamura T (2022). Aetiology and incidence of diarrhoea requiring hospitalisation in children under 5 years of age in 28 low-income and middle-income countries: findings from the Global Pediatric Diarrhea Surveillance network. BMJ Global Health.

